# Non-linear associations between blood glucose, blood lipids and inflammatory markers and new-onset arthritis in the middle-aged and older population - a cohort study in Europe

**DOI:** 10.1186/s12944-025-02495-9

**Published:** 2025-03-01

**Authors:** Fanji Qiu, Jinfeng Li, Kirsten Legerlotz

**Affiliations:** 1 Movement Biomechanics, Institute of Sport Sciences, Humboldt-Universität zu Berlin, Unter den Linden 6, 10099 Berlin, Germany; 2 Department of Kinesiology, Iowa State University, Ames, IA 50011 USA; 3 Department of Movement and Training Sciences, Institute of Sport Sciences, University of Wuppertal, Gaußstraße 20, 42119 Wuppertal, Germany

**Keywords:** Arthritis, Lipids, Inflammation, Osteoarthritis, Patient reported outcome measures

## Abstract

**Objective:**

The arthritis burden increases with aging, while blood glucose, lipid profiles and inflammatory markers may affect the development of arthritis. This study aims to determine the associations between blood markers and rheumatoid arthritis (RA) and osteoarthritis (OA) for better arthritis management.

**Method:**

Data from the 6th and 9th wave of the Survey of Health, Ageing, and Retirement in Europe (SHARE) were used. Logistic regression and Cox proportional hazards regression models were used to examine the associations between blood markers and arthritis. Generalized additive models and restricted cubic splines (RCS) were employed to assess non-linear associations.

**Results:**

This study included a total of 14,276 participants. The incidence was 5.80% for OA, and 13.92% for RA. The participants with new-onset OA and RA were more likely to be older, female, and with higher body mass index. The generalized additive model detected nonlinear associations between the incidence of OA and glycated hemoglobin A (HbA1c), and between the incidence of RA and high-density lipoprotein (HDL) and triglycerides (TRG). RCS curves (*P-nonlinear* < 0.05) showed an increased risk of new-onset OA for HbA1c levels between 4.75% and 5.91% in individuals aged ≤ 65. For those aged>65, HDL levels between 44.99 and 67.42 mg/dL and TRG levels between 265.37 and 1125.06 mg/dL were associated with an increased risks of new-onset RA. Furthermore, total cholesterol, HbA1c, HDL and TRG were associated with the prevalence of arthritis.

**Conclusion:**

Monitoring lipid profiles and HbA1c levels in middle-aged and older adults may help to manage arthritis.

**Supplementary Information:**

The online version contains supplementary material available at 10.1186/s12944-025-02495-9.

## Introduction

Arthritis encompasses a group of diseases that affect the joints and surrounding tissues, with primary symptoms including joint pain, stiffness, and swelling. Arthritis is one of the leading causes of disability worldwide, significantly impacting patients’ quality of life and work capacity. Common types of arthritis include osteoarthritis (OA) and rheumatoid arthritis (RA) [1, 2]. Osteoarthritis, the most prevalent form, primarily affects weight-bearing joints such as the knees and hips. Its pathological characteristics include degenerative changes in the articular cartilage, subchondral bone sclerosis, and mild synovial inflammation [3, 4]. Although rheumatoid arthritis also leads to joint dysfunction, its pathogenesis differs as it is an autoimmune disease typically affecting small joints in the hands and wrists, often accompanied by a systemic inflammatory responses [5]. The pathology of RA involves the immune system attacking the synovium, resulting in inflammation and destruction of the articular cartilage [6]. Factors such as higher age, mechanical injury, and metabolic disorders are considered significant contributors to the development of arthritis [7, 8], while changes in blood markers may also play a crucial role in the diagnosis and treatment of arthritis.

It is concerning that there are already approximately 40 million arthritis patients in Europe alone [9], with a prevalence rate of 31.4% among individuals over 50 years old [10]. As the population ages, by the year 2050, the number of Europeans aged 55 and above is expected to reach around 150 million, and therefore the number of arthritis patients is predicted to increase [9, 11, 12]. Additionally, there is a significant difference in the prevalence of arthritis across different age groups, ranging from 3.6% among adults aged 18–34 to 53.9% among those aged 75 and older [13]. Therefore, understanding the impact of different blood marker levels on the incidence of arthritis in middle-aged and older populations is crucial for addressing the public health challenges arthritis poses.

Numerous studies have focused on evaluating the efficacy of various treatments for arthritis patients, not only observing symptom relief and functional improvement but also noting significant changes in certain blood markers post-treatment. The disease activity score (DAS28) and the modified Health Assessment Questionnaire (M-HAQ) scores were significantly correlated with C-reactive protein (CRP), hemoglobin, and total cholesterol (TC), which was found in a cross-sectional study of 140 RA patients with a mean age of 50.3 years. Additionally, patients with longer disease duration had higher TC levels [14]. Furthermore, improvements in the European League Against Rheumatism (EULAR) scores were accompanied by significant reductions in triglycerides (TRG), TC, and low-density lipoprotein cholesterol (LDL), and increases in high-density lipoprotein cholesterol (HDL), and high-sensitivity CRP levels in RA patients after treatment [15]. Also, reductions in DAS28, CRP, and erythrocyte sedimentation rate (ESR) were associated with increases in HDL, LDL, and TC as found in a cohort study with 416 early RA patients treated with medication and followed up for 102 weeks [16]. Those studies show that associations may exist between RA and changes in blood markers.

Intriguingly, biomarker-disease relationships may exhibit complex nonlinear patterns. An example for U-shaped nonlinear relationships is the ratio of non-high-density lipoprotein cholesterol to high-density lipoprotein cholesterol (NHHR) and the prevalence of hyperuricemia, which was observed in a cross-sectional study involving 30,937 subjects aged 20 and above [17]. Nonlinear associations have also been observed between fasting glucose and CRP levels with lung function in a cross-sectional study involving 8,584 participants aged 7–79 years [18]. A prospective cohort study of 7,766 participants aged ≥ 65 years found a nonlinear relationship between HDL-C and both all-cause and cardiovascular mortality, with an optimal HDL-C range of 61 to 87 mg/dL [19]. Similar nonlinear relationship has also been observed in arthritis studies. One study based on NHANES data has found a U-shaped association between sleep duration and the incidence of OA in adults, indicating that both insufficient and excessive sleep may increase the risk of OA [20]. Additionally, another study using the same database discovered that among arthritis patients aged 19 and older, both too low and too high serum folate levels were associated with an increased risk of cancer-related mortality [21]. Therefore, one may assume that there may also be nonlinear associations between blood markers and the incidence of arthritis.

The objective of this study is therefore to evaluate the association between lipid markers (such as TC, HDL-C, TRG), inflammatory factors (such as CRP), and blood glucose indicators (such as HbA1c) and the incidence of new-onset arthritis. We hypothesize that there are nonlinear associations between blood markers and new-onset arthritis.

## Method

### Study population and design

Our cohort study utilized data from 6th and 9th wave of the Survey of Health, Ageing, and Retirement in Europe (SHARE). SHARE is a large-scale longitudinal research database based on the European population, encompassing multidisciplinary information on approximately 140,000 adults aged 30 and above, including their families, covering information on health and socioeconomic status [22–24]. Since its inception in 2004, SHARE has released nine waves of data biennially. Wave 6 was completed in 2015, and Wave 9 in 2021, with each data collection using standardized questionnaires [25]. SHARE has obtained approval from relevant ethics committees in all participating countries, and all participants provided written informed consent [23].

**Fig. 1:**
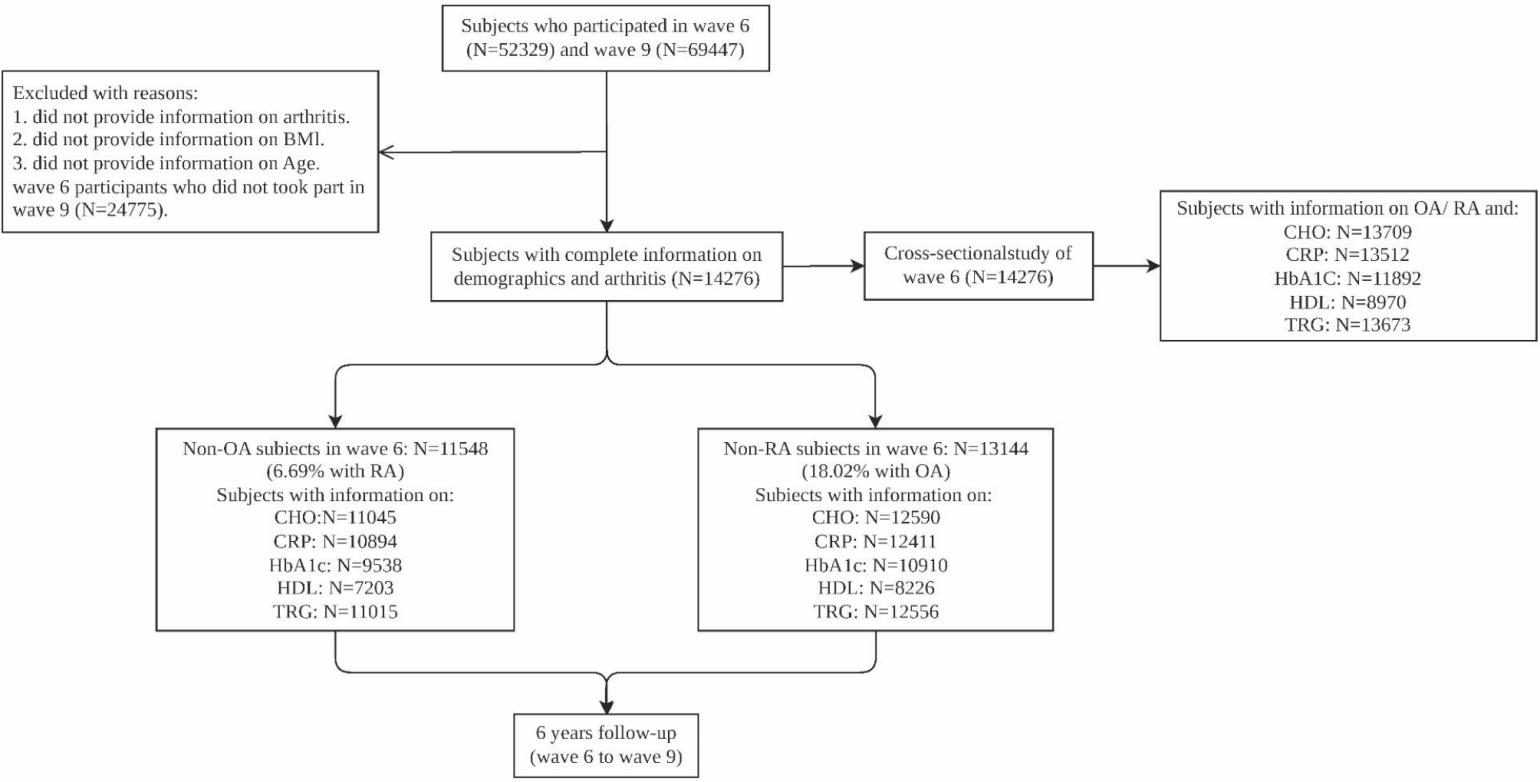
Flow diagram. Abbreviation: BMI: body mass index; CHO: total cholesterol; CRP: C-reactive protein; HbA1c: glycated hemoglobin A; HDL: high density lipoprotein; TRG: triglycerides

This study included both a cross-sectional as well as a longitudinal experimental design. The cross-sectional study explored the association between blood markers and arthritis at Wave 6, while the longitudinal study examined the association between blood marker levels and the new-onset of new arthritis cases six years later. A total of 27,554 community-dwelling residents were included in the study (Fig. 1). All participants provided information on age, BMI, and sex to avoid potential biases caused by missing data.

### Demographic and health-related information

Demographic and health-related information was collected from participants through standardized questionnaires. Demographic data included age (categorized as middle-aged: ≤65 and older: >65, to explore the association between blood markers and new-onset arthritis in different age groups), body mass index (BMI), and sex. Health-related information included the presence of rheumatoid arthritis or osteoarthritis. Due to the setting of the questionnaire, osteoarthritis and other rheumatic diseases were grouped together.

### Blood markers

Blood markers were collected in wave 6, with 25,248 participants providing blood marker information. The blood markers of interest included total cholesterol (CHO), C-reactive protein (CRP), glycated hemoglobin A (HbA1c), high-density lipoprotein (HDL), and triglycerides (TRG).

### Statistical analysis

Data analysis was conducted using Python and SPSS 26.0 (IBM: Armonk, NY, USA). Based on prevalence rates of 19.32% for OA and 10.19% for RA [26], a 95% confidence interval and a β error of 5%, the minimum required sample size for this study was calculated to be 240 [27]. Descriptive analyses characterized the baseline features of the study population: (1) age, (2) BMI, (3) sex, and levels of blood markers, with means and standard deviations (SD) reported. Continuous data that is not normally distributed is presented using the median and interquartile range (IQR). Chi-square tests or independent sample t-tests were used to explore differences between those with and without disease, as well as between middle-aged and older participants.

Binary logistic regression and Cox regression were employed to evaluate the cross-sectional and longitudinal associations between independent variables (blood markers), covariates (age, BMI, sex), and dependent variables (RA and OA), with a follow-up period of 6 years for Cox regression. Odds ratios (OR), hazard ratios (HR), 95% confidence intervals, and *P* values were calculated. A *P* value of < 0.05 was considered statistically significant. The category set as the reference category was male sex. The analysis of binary logistic regression and Cox regression was conducted in two steps: first, a crude analysis where independent variables were entered into the model; second, an adjusted analysis where both independent variables and covariates were included in model. Age, sex, and BMI had *p* < 0.05 in univariate tests, and these covariates were identified as potential confounders. The missing BMI in wave 9 was imputed with the mean. A generalized additive model was applied to explore the nonlinear associations between the independent variables and the HR for the two types of arthritis, and a restricted cubic spline (RCS) based on the Cox hazards model was plotted, with five knots at the 5th, 27.5th, 50th, 72.5th, and 95th percentiles of the independent variables [28]. Sensitivity analyses based on obesity classes were also performed to determine whether the presence of obesity affected the robustness of the results. Multicollinearity was assessed to detect collinearity in the regression models, with strong collinearity defined by a variance inflation factor (VIF) greater than 5 and a tolerance value less than 0.20 [29, 30].

## Results

### Participant characteristics

A total of 27,554 subjects participated in both wave 6 and wave 9. After excluding those with missing health information, 14,276 were included in the final cross-sectional and longitudinal analysis. The average age of all included subjects was 66.15 ± 8.62 years, the average BMI was 27.03 ± 4.56, and females accounted for 58.90% (Table 1). The collinearity diagnostics did not reveal any collinearity among the variables.

**Table 1:** The characteristics of participants (wave 6)

	ALL	OA			RA		
		With	Without	*P*-value	With	Without	*P*-value
*N * **(%)**	14,276	2728 (19.11)	11,548 (80.89)		1132 (7.92)	13,144 (92.07)	
**Age**	66 (60–72)	67 (61–74)	65 (59–72)	<0.001	69 (62–75)	65 (59–72)	<0.001
**BMI**	26.45 (23.94–29.41)	27.06 (24.22–30.12)	26.30 (23.88–29.36)	<0.001	28.08 (25.20–31.60)	26.32 (23.88–29.37)	<0.001
**Female**	8409 (58.90)	1966 (23.40)	6443 (76.60)	<0.05	838/ 8409	7571/ 8409	<0.05

Abbreviation: BMI: body mass index; OA: osteoarthritis; RA: rheumatoid arthritis

Age and BMI are presented as median and IQR due to non-normal distribution. Female is presented as number (%) or female / (female + male). Wave 6: the 6th data collection wave of SHARE

The cross-sectional analysis of wave 6 data revealed 19.11% participants with OA and 7.92% participants with RA. Analysis of blood markers showed that the CRP and HbA1c levels of the older population were significantly higher than those of the middle-aged population (*P*<0.05), while the CHO, HDL and TRG levels of the middle-aged population were significantly higher than those of the older population (*P*<0.05) (Table 2). OA patients had higher CRP, HDL and TRG levels than OA-free participants (*P*<0.05). Compared with RA-free participants, RA patients had lower CHO and HDL levels and higher CRP, HbA1c and TRG levels (*P*<0.05).

**Table 2:** The blood markers of participants (wave 6)

Variables	Middled-aged ≤ 65 years	Older > 65 years	*P*-value	OA			RA		
				With	Without	*P*-value	With	Without	*P*-value
**CHO (**mg/dL**)**	226.39 (210.95-242.71)	222.63 (206.86-238.96)	<0.001	225.29 (209.10-241.42)	224.38 (208.52-240.63)	>0.05	222.36 (206.86-238.26)	224.81 (208.77-241.03)	<0.05
**CRP (**mg/L**)**	1.98 (1.44–3.03)	2.07 (1.50–3.11)	<0.001	2.17 (1.56–3.35)	2.00 (1.45–3.01)	<0.001	2.27 (1.61–3.74)	2.01 (1.46–3.02)	<0.001
**HbA1c (**%**)**	5.88 (5.65–6.16)	5.98 (5.75–6.26)	<0.001	5.94 (5.70–6.20)	5.93 (5.70–6.22)	>0.05	5.99 (5.76–6.28)	5.93 (5.69–6.21)	<0.001
**HDL (**mg/dL**)**	68.70 (62.87–75.12)	67.85 (62.33–73.89)	<0.001	68.83 (63.49–73.04)	68.12 (62.39–74.33)	<0.001	66.15 (60.74–72.36)	68.48 (62.83–74.72)	<0.001
**TRG (**mg/dL**)**	199.84 (148.33-270.97)	196.72 (148.03-263.99)	<0.05	204.02 (151.27-273.83)	197.12 (147.26-265.78)	<0.05	209.40 (158.00-278.76)	197.36 (147.28-265.96)	<0.05

Abbreviation: CHO: total cholesterol; CRP: C-reactive protein; HbA1c: Hemoglobin A1c; HDL: high-density lipoprotein; TRG: triglycerides

Data are presented as median and IQR due to non-normal distribution

During wave 6, there were 11,548 participants identified as OA-free and 13,144 participants identified as RA-free who also participated in the data collection for wave 9. About 13.92% baseline OA-free participants developed OA and 5.80% baseline RA-free participants developed RA over the 6 year study period. Compared to participants without arthritis, participants with OA and RA in wave 6 and new-onset OA and RA in wave 9 were more likely to be older, female, and to have a higher BMI.

### Risk of new-onset arthritis

**Fig. 2:**
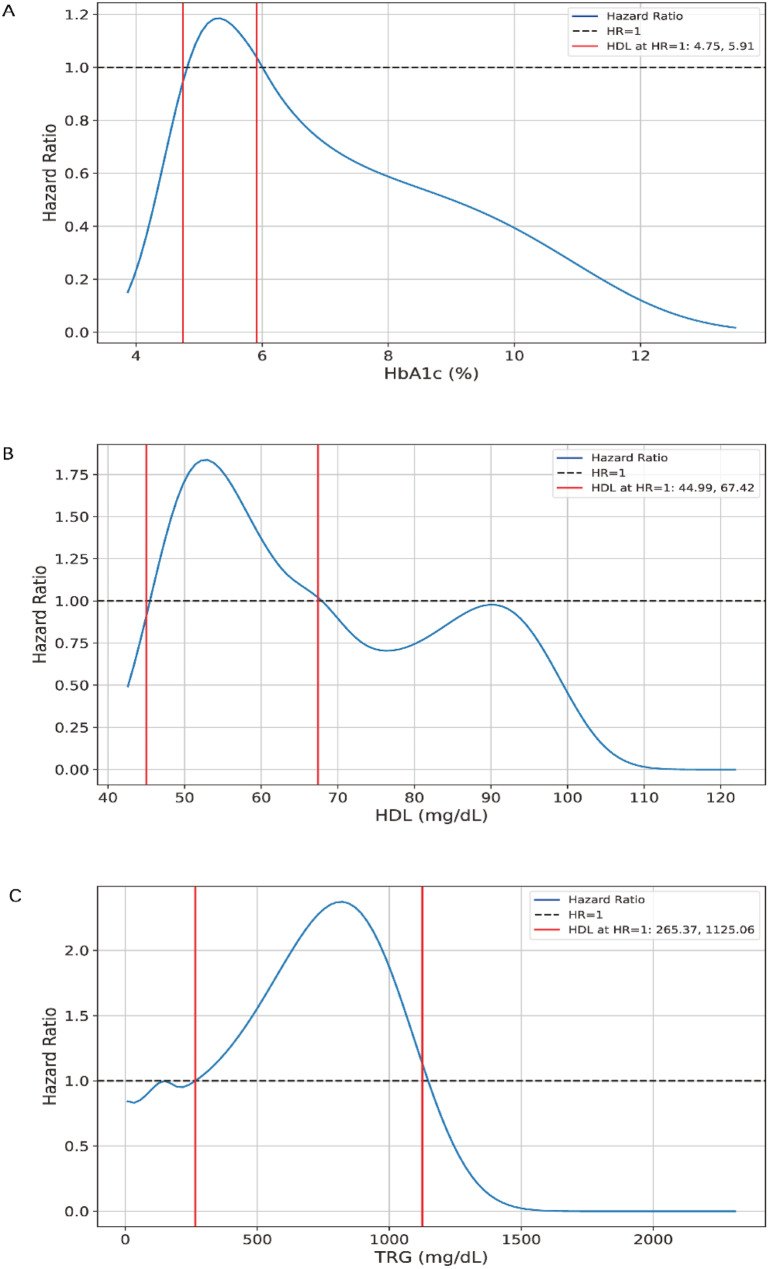
Restricted cubic spline regression analysis of blood markers with HR of arthritis. The reference line for no association is represented by the dashed black line, with a hazard ratio of 1.0. **A**: restricted cubic spline for HbA1c, in participants aged ≤ 65 years, the nonlinear association was observed between OA and HbA1c (*P-nonlinear* < 0.005). **B** and **C**: restricted cubic spline for HDL and TRG in participants aged > 65 years, nonlinear associations were observed between RA and HDL (*P-nonlinear* < 0.005) and between RA and TRG (*P-nonlinear* = 0.04). Abbreviation: HR: hazard ratio

After stratifying by age (Table 3), the adjusted cox regression model revealed that in participants aged ≤ 65 years the HbA1c level was significantly associated with the risk of new-onset OA (HR = 0.760, *P* < 0.05). Among participants aged over 65 years, significant associations were found between HDL (HR = 0.760, *P* < 0.05), TRG (HR = 0.760, *P* < 0.05) and the risk of new-onset RA. The generalized additive model observed a nonlinear association between the OA incidence and HbA1c in participants aged ≤ 65 years (*P*-*nonlinear* < 0.005). In participants aged > 65 years, nonlinear associations were observed between RA incidence and HDL (*P*-*nonlinear* < 0.005) and between RA incidence and TRG (*P*-*nonlinear* = 0.04). The RCS curves (Fig. 2) indicated that the risk of developing new-onset OA increased for participants aged ≤ 65 years when HbA1c levels were between 4.75% and 5.91%. For participants aged > 65 years, the risk of developing RA increased when HDL levels were between 44.99 and 67.42 mg/dL and when TRG levels were between 265.37 and 1125.06 mg/dL.

**Table 3:** Hazard ratio (HRs) and 95% confidence interval (CI) of new-onset arthritis in relation to baseline characteristics in different age groups

		OA						RA			
Variables	Age group	*N*	HR-Crude	*P*	HR-Adjusted	*P*	*N*	HR-Crude	*P*	HR-Adjusted	*P*
**CHO**	≤ 65	5688	1.000 (0.997–1.003)	0.751	1.000 (0.997–1.003)	0.835	6354	0.998 (0.994–1.003)	0.548	0.999 (0.994–1.004)	0.827
	>65	5357	1.001 (0.998–1.004)	0.465	1.000 (0.998–1.003)	0.812	6236	0.997 (0.993–1.001)	0.114	0.997 (0.993-1.000)	0.077
**CRP**	≤ 65	5620	1.000 (0.985–1.015)	0.974	0.993 (0.975–1.010)	0.401	6270	1.010 (0.991–1.030)	0.308	1.001 (0.978–1.025)	0.935
	>65	5274	1.006 (0.995–1.016)	0.301	1.004 (0.993–1.015)	0.450	6141	1.013 (1.002–1.024)	0.023	1.010 (0.998–1.022)	0.115
**HbA1c**	≤ 65	4956	0.819 (0.696–0.964)	0.016	0.760 (0.640–0.902)	0.002	5546	1.128 (0.909–1.398)	0.274	1.052 (0.834–1.328)	0.668
	>65	4582	0.938 (0.822–1.070)	0.343	0.906 (0.790–1.040)	0.161	5364	1.194 (1.048–1.360)	0.008	1.133 (0.987–1.301)	0.077
**HDL**	≤ 65	3771	1.007 (0.998–1.017)	0.129	1.009 (0.999–1.019)	0.076	4217	0.994 (0.978–1.010)	0.431	0.996 (0.979–1.012)	0.611
	>65	3432	1.005 (0.996–1.015)	0.247	1.006 (0.997–1.015)	0.204	4009	0.971 (0.958–0.984)	<0.001	0.973 (0.959–0.986)	<0.001
**TRG**	≤ 65	5674	1.000 (1.000-1.001)	0.427	1.000 (1.000-1.001)	0.701	6340	1.000 (0.999–1.001)	0.648	1.000 (0.999–1.001)	0.959
	>65	5341	1.000 (1.000-1.001)	0.382	1.000 (1.000-1.001)	0.756	6216	1.001 (1.000-1.001)	0.004	1.001 (1.000-1.001)	0.045

Data presented as HR (corresponding 95% CI)

Adjusted Cox regression model was adjusted for co-variates: age (years), sex (male, female), BMI (kg/m^2^)

Sensitivity analysis (Supplemental File) revealed the robustness of the nonlinear associations. Among individuals over 65 years, the incidence of RA demonstrated significant associations with cholesterol levels (HR = 0.996, *P* < 0.05) and HbA1c levels (HR = 1.168, *P* < 0.05). In the ≤ 65-year cohort, RA prevalence was associated with CRP levels (HR = 1.015, *P* < 0.05).

### Associations between blood markers and arthritis

The cross-sectional analysis revealed that for participants aged ≤ 65 years (Table 4), HbA1c levels were negatively associated with OA prevalence (*P*<0.05), while TRG levels were positively associated with OA prevalence (*P*<0.05). HDL levels were negatively associated with RA prevalence. In participants aged over 65 years, CHO levels were negatively associated with RA prevalence (*P*<0.05), and HDL levels were negatively associated with RA prevalence but positively associated with OA prevalence (*P*<0.05).

**Table 4:** Results of binary logistic regression in different age groups

			OA					RA		
Variables	Age group	*N*	OR-Crude	*P*	OR-Adjusted	*P*	OR-Crude	*P*	OR-Adjusted	*P*
**CHO**	≤ 65	6789	1.000 (0.997–1.002)	0.924	1.000 (0.997–1.003)	0.912	0.998 (0.994–1.002)	0.226	0.999 (0.995–1.003)	0.609
	>65	6920	1.003 (1.000-1.005)	0.018	1.001 (0.999–1.004)	0.267	0.995 (0.992–0.999)	0.006	0.995 (0.992–0.998)	0.003
**CRP**	≤ 65	6700	1.011 (1.000-1.022)	0.044	1.004 (0.992–1.016)	0.527	1.021 (1.008–1.033)	0.001	1.011 (0.997–1.026)	0.136
	>65	6812	1.008 (0.999–1.017)	0.090	1.006 (0.996–1.015)	0.241	1.014 (1.003–1.025)	0.012	1.010 (0.998–1.022)	0.089
**HbA1c**	≤ 65	5941	0.858 (0.748–0.984)	0.028	0.773 (0.666–0.896)	0.001	1.264 (1.079–1.480)	0.004	1.140 (0.956–1.358)	0.144
	>65	5953	0.947 (0.850–1.056)	0.326	0.916 (0.817–1.026)	0.129	1.060 (0.919–1.222)	0.425	0.970 (0.831–1.133)	0.699
**HDL**	≤ 65	4509	1.004 (0.996–1.013)	0.320	1.006 (0.997–1.015)	0.192	0.981 (0.968–0.994)	0.005	0.986 (0.972-1.000)	0.044
	>65	4461	1.018 (1.010–1.026)	<0.001	1.017 (1.009–1.025)	<0.001	0.966 (0.955–0.978)	<0.001	0.968 (0.956–0.979)	<0.001
**TRG**	≤ 65	6774	1.001 (1.000-1.001)	0.006	1.000 (1.000-1.001)	0.027	1.001 (1.000-1.001)	0.032	1.000 (1.000-1.001)	0.153
	>65	6899	1.001 (1.000-1.001)	0.014	1.000 (1.000-1.001)	0.140	1.000 (1.000-1.001)	0.408	1.000 (0.999–1.001)	0.842

Data presented as OR (corresponding 95% CI)

Adjusted logistic regression model was adjusted for co-variates: age (years), sex (male, female), BMI (kg/m^2^)

## Discussion

In this large cohort study of middle-aged and older adults, we observed significant associations between HbA1c levels and new-onset OA, and between HDL and TRG levels and new-onset RA. Additionally, significant associations between higher HDL and TRG levels with increased OA prevalence, lower HbA1c levels with decreased OA prevalence, and lower lipid levels (CHO, HDL, TRG) with decreased RA prevalence were revealed.

Our study highlights the association between lipid levels and arthritis, and further uncovers nonlinear associations with new-onset RA. The influence of lipid profiles on RA clinical outcomes has been previously noted. The lipid profile characterized by high TRG and low HDL was associated with systemic inflammation and poor clinical outcomes in RA patients’ post-treatment. This association is potentially due to the dysfunctional metabolism of TRG-rich lipoproteins and HDL [31]. The RA patients exhibited an adverse lipid profile—low HDL and high TRG—compared to healthy individuals, possibly associated to insulin resistance, which was revealed in a controlled trial of 180 subjects aged 24–68 [32]. Similar findings were reported in a cohort study of 577 RA patients with an average age of 56.8 years, where total cholesterol levels were significantly lower in the RA cohort compared to non-RA subjects during the first five years of RA onset [33]. This reduction might be due to decreased plasma cholesterol concentration under inflammatory conditions [34], or increased catabolism of cholesterol [35]. Early assessment of lipid levels could thus aid in better management and prevention of RA. On the other hand, we also observed that HDL was a risk factors for OA. This has been described previously as HDL has been identified as risk factors in a cross-sectional analysis in adults [36]. Our findings indicated that middle-aged individuals have higher HDL level compared to older adults. Despite the higher prevalence and incidence of OA with increasing age, middle-aged individuals should also monitor their HDL levels to manage the risk of OA.

Our study also highlights the differential impact of lipid levels on various types of arthritis. Our findings indicate that HDL was associated with a higher prevalence of OA, possibly due to excessive lipid deposition in osteoarthritic chondrocytes, disrupting the balance between lipogenesis and chondrogenesis [37]. However, we did not observe a significant association between lipid levels and new-onset OA, consistent with a cohort study of 1,512 subjects with an average age of 62, which found no association between cholesterol, low-density lipoprotein, or high-density lipoprotein and the new-onset of knee osteoarthritis over a five-year period [38]. For RA, significant associations were observed between CHO, HDL, TRG levels and both the prevalence and incidence of RA in our study. The anti-inflammatory function of HDL was negatively correlated with systemic inflammation in RA patients, which was found in a cross-sectional study of 132 RA patients with an average age of 53 [39]. Additionally, lipid peroxidation of HDL may impair the anti-inflammatory, antioxidant, and cholesterol efflux capacities, promoting LDL oxidation and accelerating atherosclerosis in RA patients. Thus, higher HDL levels may help maintain normal lipid metabolism in RA patients [39]. We also observed that CHO and HDL levels were lower in RA patients compared to non-RA subjects, while TRG levels were higher in RA patients. The reduction in CHO levels in RA patients may be related to the decrease in HDL [40]. An analysis of lipid profiles in 79 subjects who later developed RA found that TRG levels were 17% higher compared to healthy controls, possibly due to elevated inflammation levels [41]. Therefore, RA patients can potentially prevent and manage RA by monitoring and adjusting their HDL and TRG levels.

The association between HbA1c and OA is discussed controversially, and our findings offer new insights. We observed an increased risk of new-onset OA in individuals aged ≤ 65 years when HbA1c levels were between 4.75% and 5.91%. Similar results were found in a cross-sectional study of 85 knee OA patients aged 19 to 86, where the risk of disease increased by 1.7% for each unit increase in blood glucose [7]. However, inconsistent results have been reported in cohort studies. For instance, no association between blood serum HbA1c levels and the incidence or progression of OA was found a cohort study of 1,384 subjects with an average age of 63.9 years over a three-year follow-up [42]. No association between impaired fasting glucose and the incidence of total knee replacement due to OA was observed in another cohort study involving 1,222 patients aged 27 to 75 who underwent joint replacement surgery due to OA over an average follow-up of 6.8 years [43]. Compared to these studies, our OA patients had a higher BMI of 27 and a median age of 67 years. Additionally, these studies adjusted for covariates such as race, education, height, and total body fat, which may account for the differences in findings. In contrast, a cross-sectional study of 6,197 participants aged 45–65 found a weak negative correlation between HbA1c levels and the presence of OA in the hands and knees among males while they detected no association in weight-bearing joints such as the knee [44]. Furthermore, a meta-analysis including 863,755 subjects found that diabetes reduced the future risk of gout, with a stronger protective effect observed in males, those with type 1 diabetes, and those with higher HbA1c levels. This protective effect may be due to the substantial role of the uricosuric effect of glycosuria and the impaired inflammatory response [45]. Therefore, the development and onset of OA are influenced by multiple factors, and even when HbA1c levels are within the normal range, active arthritis management is necessary.

Notably, obesity may play a critical role in influencing arthritis health. As a multifactorial metabolic disorder, obesity contributes to various health complications and demonstrates strong associations with systemic metabolic dysregulation, including dyslipidemia and impaired glucose metabolism [46]. These metabolic perturbations may further exacerbate arthritis pathogenesis. Comparative studies in middle-aged and older populations reveal that obesity correlates with more severe synovitis and structural abnormalities in knee OA, while the co-occurrence of obesity and hyperlipidemia exhibits synergistic detrimental effects on knee OA progression [47]. In rheumatoid arthritis populations, obesity is not only frequently observed but also associated with significantly reduced physical health scores [48]. Our study demonstrates that 22.2% of participants met obesity criteria, highlighting the necessity of weight management interventions in aging populations to mitigate arthritis burden.

There are some limitations in our study. Firstly, while the SHARE database has considered population representativeness in its initial sampling, this representativeness may diminish after data filtering and the exclusion of cases with missing data. Secondly, although demographic factors were accounted for in our statistical models, other confounding factors, such as education level, may still influence the results. Lastly, due to the limitations in the questionnaire setting, OA was grouped with other forms of rheumatism, potentially biasing the prevalence and incidence results for OA.

## Conclusion

This cohort study found significant associations between HbA1c and new-onset OA, and between HDL and TRG levels and new-onset RA over a six-year follow-up. For individuals aged ≤ 65 years, the risk of new-onset OA increased when HbA1c levels were between 4.75% and 5.91%. For those aged > 65 years, the risk of new-onset RA increased when HDL levels were between 44.99 and 67.42 mg/dL, and TRG levels were between 265.37 and 1125.06 mg/dL. Cross-sectional analysis also revealed a negative association between HDL levels and RA prevalence, and between HbA1c levels and OA prevalence in middle-aged individuals. Therefore, monitoring changes in lipid and HbA1c levels in middle-aged and older adults may help to manage arthritic diseases.

## Electronic supplementary material

Below is the link to the electronic supplementary material.

Supplementary Material 1

## Data Availability

No datasets were generated or analysed during the current study.

## Abbreviations

CAD: Coronary artery disease
CRP: C-reactive protein
DAS28: Disease activity score
EULAR: European League Against Rheumatism scores
HAQ-DI: The Health Assessment Questionnaire-Disability Index
HDL: High-density lipoprotein cholesterol
HR: Hazard ratios
ICOAP: Intermittent and Constant Osteoarthritis Pain
IQR: Interquartile range
LDL: Low-density lipoprotein cholesterol
M-HAQ: The modified Health Assessment Questionnaire scores
OA: Osteoarthritis
OR: Odds ratios
RA: Rheumatoid arthritis
SHARE: The Survey of Health, Ageing, and Retirement in Europe
SD: Standard deviations
TC: Total cholesterol
TRG: Triglycerides
TyG: Triglyceride-glucose index
VAS: Visual Analog Scale for Pain
WOMAC: The Western Ontario and McMaster Universities Osteoarthritis Index
