# Supplementary material for: Non-linear associations between blood glucose, blood lipids and inflammatory markers and new-onset arthritis in the middle-aged and older population - a cohort study in Europe
Source: Lipids Health Dis. 2025 Mar 1;24:79. doi: 10.1186/s12944-025-02495-9 (PMC11872311; doi:10.1186/s12944-025-02495-9)
Supplement: Supplementary file 1 — Supplementary Material 1 [file 12944_2025_2495_MOESM1_ESM.docx]

**Demographic information of subjects (2021)**

OA

With OA: 27.20±5.04, OA-free: 26.62±4.51

RA

With RA: 27.78±5.25, RA-free: 26.62±4.57

**Description of Variables**

THB [g/dL]

HDL [mg/dL]

CHO [mg/dL]

CRP [mg/L]

cyc [mg/L]

TRG [mg/dL]

HbA1c [%]

CRP，OA or non-OA participants grouping by age:

|  | ≤65 | ＞65 | P-value |
| --- | --- | --- | --- |
| non-OA | 5.9832 (0.55953) | 6.0782 (0.59471) | ﹤0.001 |
| OA | 5.9236 (0.46001) | 6.0537 (0.53967) | ﹤0.001 |

CHO: OA or non-OA participants grouping by age:

|  | ≤65 | ＞65 | P-value |
| --- | --- | --- | --- |
| non-OA | 226.4658 (24.77) | 222.0775 (24.32) | ﹤0.001 |
| OA | 226.1273 (24.79) | 222.8059 (24.73) | ﹤0.01 |

Obesity%：22.2%

**Sensitivity analyses (Table 1, 2, figure 1)**

Table 1. results of COX regression (BMI → Obesity classes)

| **OA** |  | **P** | **HR-adjusted** | **95.0% CI lower limit** | **95.0% CI upper limit** | **RA** |  | **P** | **HR-adjusted** | **95.0% CI lower limit** | **95.0% CI upper limit** |
| --- | --- | --- | --- | --- | --- | --- | --- | --- | --- | --- | --- |
| **CHO** | ≤65 | 0.752 | 1 | 0.997 | 1.003 | **CHO** | ≤65 | 0.629 | 0.999 | 0.994 | 1.004 |
|  | ﹥65 | 0.908 | 1 | 0.997 | 1.003 |  | ﹥65 | 0.042 | 0.996 | 0.992 | 1 |
| **CRP** | ≤65 | 0.534 | 0.995 | 0.978 | 1.011 | **CRP** | ≤65 | 0.672 | 1.005 | 0.983 | 1.026 |
|  | ﹥65 | 0.442 | 1.004 | 0.993 | 1.015 |  | ﹥65 | 0.079 | 1.011 | 0.999 | 1.023 |
| **HbA1c** | ≤65 | 0.003 | 0.774 | 0.653 | 0.918 | **HbA1c** | ≤65 | 0.475 | 1.087 | 0.865 | 1.364 |
|  | ﹥65 | 0.195 | 0.913 | 0.796 | 1.048 |  | ﹥65 | 0.026 | 1.168 | 1.018 | 1.34 |
| **HDL** | ≤65 | 0.14 | 1.007 | 0.998 | 1.017 | **HDL** | ≤65 | 0.481 | 0.994 | 0.978 | 1.011 |
|  | ﹥65 | 0.277 | 1.005 | 0.996 | 1.014 |  | ﹥65 | 0 | 0.97 | 0.957 | 0.983 |
| **TRG** | ≤65 | 0.587 | 1 | 1 | 1.001 | **TRG** | ≤65 | 0.821 | 1 | 0.999 | 1.001 |
|  | ﹥65 | 0.703 | 1 | 1 | 1.001 |  | ﹥65 | 0.025 | 1.001 | 1 | 1.001 |

Table 2. results of logistic regression (BMI → Obesity classes)

| **OA** |  | **P** | **OR-adjusted** | **95.0% CI lower limit** | **95.0% CI upper limit** | **RA** |  | **P** | **OR-adjusted** | **95.0% CI lower limit** | **95.0% CI upper limit** |
| --- | --- | --- | --- | --- | --- | --- | --- | --- | --- | --- | --- |
| **CHO** | ≤65 | 0.842 | 1 | 0.997 | 1.002 | **CHO** | ≤65 | 0.359 | 0.998 | 0.994 | 1.002 |
|  | ﹥65 | 0.431 | 1.001 | 0.999 | 1.003 |  | ﹥65 | 0.001 | 0.994 | 0.991 | 0.998 |
| **CRP** | ≤65 | 0.39 | 1.005 | 0.993 | 1.017 | **CRP** | ≤65 | 0.034 | 1.015 | 1.001 | 1.028 |
|  | ﹥65 | 0.237 | 1.006 | 0.996 | 1.015 |  | ﹥65 | 0.053 | 1.011 | 1 | 1.023 |
| **HbA1c** | ≤65 | 0.003 | 0.804 | 0.695 | 0.93 | **HbA1c** | ≤65 | 0.055 | 1.182 | 0.996 | 1.401 |
|  | ﹥65 | 0.225 | 0.933 | 0.833 | 1.044 |  | ﹥65 | 0.91 | 1.009 | 0.866 | 1.175 |
| **HDL** | ≤65 | 0.449 | 1.003 | 0.995 | 1.012 | **HDL** | ≤65 | 0.009 | 0.982 | 0.968 | 0.995 |
|  | ﹥65 | 0 | 1.015 | 1.007 | 1.023 |  | ﹥65 | 0 | 0.965 | 0.954 | 0.977 |
| **TRG** | ≤65 | 0.014 | 1.001 | 1 | 1.001 | **TRG** | ≤65 | 0.092 | 1.001 | 1 | 1.001 |
|  | ﹥65 | 0.085 | 1 | 1 | 1.001 |  | ﹥65 | 0.948 | 1 | 0.999 | 1.001 |


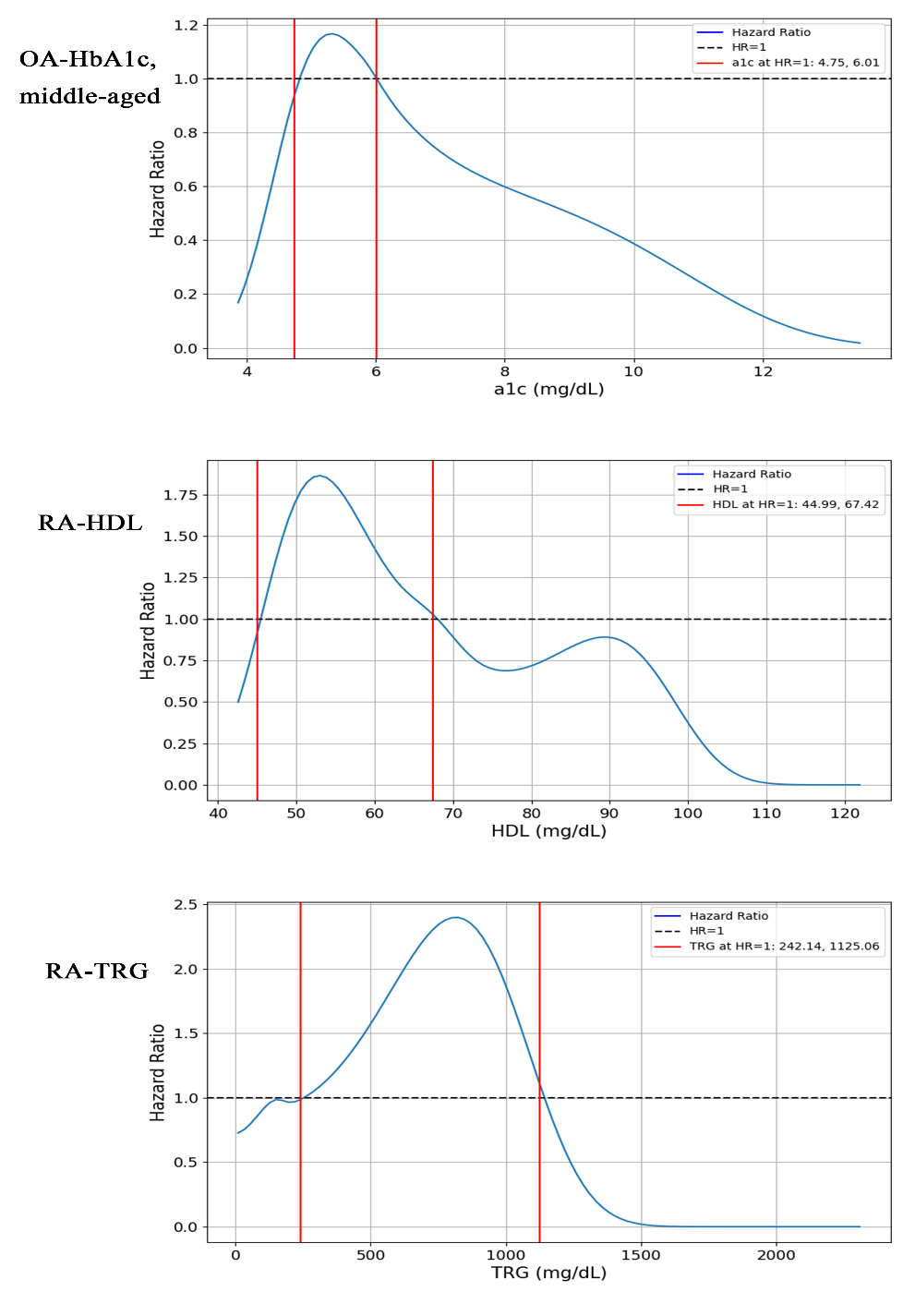


Figure 1
